# Heat Shock Responsive Gene Expression Modulated by mRNA Poly(A) Tail Length

**DOI:** 10.3389/fpls.2020.01255

**Published:** 2020-08-14

**Authors:** Xuan Wu, Jie Wang, Xiaohui Wu, Yiling Hong, Qingshun Quinn Li

**Affiliations:** ^1^ Key Laboratory of the Ministry of Education for Coastal and Wetland Ecosystem, College of the Environment and Ecology, Xiamen University, Xiamen, China; ^2^ Graduate College of Biomedical Sciences, Western University of Health Sciences, Pomona, CA, United States; ^3^ Department of Biology, Miami University, Oxford, OH, United States; ^4^ Department of Automation, Xiamen University, Xiamen, China; ^5^ College of Veterinary Medicine, Western University of Health Sciences, Pomona, CA, United States

**Keywords:** posttranscriptional regulation, polyadenylation, heat shock response, gene expression, Arabidopsis

## Abstract

Poly(A) tail length (PAL) has been implicated in the regulation of mRNA translation activities. However, the extent of such regulation at the transcriptome level is less understood in plants. Herein, we report the development and optimization of a large-scale sequencing technique called the Assay for PAL-sequencing (APAL-seq). To explore the role of PAL on post-transcriptional modification and translation, we performed PAL profiling of Arabidopsis transcriptome in response to heat shock. Transcripts of 2,477 genes were found to have variable PAL upon heat treatments. Further study of the transcripts of 14 potential heat-responsive genes identified two distinct groups of genes. In one group, PAL was heat stress-independent, and in the other, PAL was heat stress-sensitive. Meanwhile, the protein expression of HSP70 and HSP17.6C was determined to test the impact of PAL on translational activity. In the absence of heat stress, neither gene demonstrated protein expression; however, under gradual or abrupt heat stress, both transcripts showed enhanced protein expression with elongated PAL. Interestingly, HSP17.6C protein levels were positively correlated with the severity of heat treatment and peaked when treated with abrupt heat. Our results suggest that plant genes have a high variability of PALs and that PAL contributes to swift posttranslational stress responses.

## Introduction

As an essential environmental factor, temperature has far-reaching impacts on plant growth and development. This is especially true for land plants exposed to a wide range of temperature fluctuation daily and/or seasonally. Modern agriculture requires high crop productivity to meet increasing food demands. However, crop productivity can be directly affected by fluctuations in temperature (Long and Ort, 2010). Unlike animals, plants cannot flee stressful temperature conditions. Thus, even a small increase in temperature can lead to protein unfolding, entanglement, and nonspecific aggregation. Mild heat stress has been shown to result in the reorganization of actin filaments into stress-defense forms. Severe heat stress results in the aggregation of vimentin or other filament-forming proteins, leading to the collapse of actin and tubulin networks (Welch and Suhan, 1985; Welch and Suhan, 1986).

Thermotolerance is a response plant cells have when encountering unfavorable heat stress (Finka et al., 2011). A thermotolerant state might cross talk with other mechanisms of stress responses, for example, hypoxia or oxidative stress (Iba, 2002; Banti et al., 2008; Hua, 2009). Heat shock proteins (HSPs) are actively expressed and accumulated by plants in response to heat stress. Molecular mechanisms underlying HSP-mediated thermotolerance are usually performed by interactions with misfolded proteins (Parsell and Lindquist, 1993). While some HSPs induce the degradation of misfolded proteins, such as Lon, ubiquitin, and various ubiquitin-conjugating enzymes, Hsp70 and Hsp60 prevent folding intermediates from aggregating. Hsp100 can even reactivate protein complexes aggregated by misfolded proteins. However, the detailed regulatory mechanisms underlying thermotolerance remain unclear.

Posttranscriptional regulation enables plants to fine-tune their gene expression in order to properly respond to external stimuli, including heat stress. The pre-mRNA molecule undergoes three main modifications, 5’ capping, 3’ polyadenylation, and splicing, which occur in the cell nucleus before the mRNA becomes mature. Polyadenylation of mRNA is an essential step for all plants. Poly(A) tails are formed by adding a stretch of RNA with only adenine bases to the 3′-end of fully processed eukaryotic mRNA through polyadenylation. They are also actively involved in mRNA metabolism, including mRNA stability, mRNA translational efficiency, and the transportation of processed mRNA from the nucleus to the cytoplasm (Xing and Li, 2011).

Previous research, regarding some transcripts of heat shock protein-coding genes, has revealed that the length of poly(A) tails may contribute to faster heat stress responses in animals and plants. Moerman and colleagues demonstrated that the poly(A) tail of HSP32 RNA was 100 nucleotides (nt) longer under heat stress in *Dictyostelium discoideum* (Moerman et al., 1998). Only severe heat shock (above 37°C) can promote full polyadenylation of HSP70 transcripts in *Drosophila melanogaster* (Dellavalle et al., 1994). In *Arabidopsis*, the poly(A) tail length (PAL) of HSP21 increased under severe heat stress (Osteryoung et al., 1993). In general, however, PAL is poorly studied and is currently mostly limited to individual transcripts (Zhao et al., 2019). Furthermore, the contribution of PAL to plant thermotolerance remains unclear on a transcriptome level.

In this study, we developed and optimized a novel protocol to study PAL at the whole transcriptome level. Using this method, which we termed APAL-seq, PAL in the *Arabidopsis* transcriptome was determined for gradual and abrupt heat treatments. The potential roles of PAL were explored to uncover its association with HSP gene expression. We hypothesize that a change in PAL serves as the key component in the regulatory system during thermotolerance response in *Arabidopsis*.

## Materials and Methods

### *Arabidopsis* Cell Culture and Heat Shock Treatments

*Arabidopsis* (*Arabidopsis thaliana* (L.) Heynh. [ecotype Landsberg erecta (Ler)] cell suspensions (originally from the *Arabidopsis* Biological Resources Center, Columbus, Ohio, USA) were grown in 50 ml liquid medium (1x Murashige and Skoog (MS) basal salts, 1X Gamborg’s B5 vitamins, 3% [w/v] Sucrose, 0.59 g/L MES, 0.5 mg/L 1-naphthaleneacetic acid, and 0.05 mg/L benzyl aminopurine, pH 5.7) at 25°C with gentle agitation (130 rpm) in 16/8-h light/dark cycles (Zhao et al., 2011). A 6- ml aliquot was transferred to 50 ml fresh medium each week (Holdorf et al., 2012).

Both gradual and abrupt heat treatments were performed in a programmable growth chamber. For gradual heat treatment, temperature was increased from 22°C to 40°C at a rate of 1°C/15 min and then maintained at 40°C for 1h. For abrupt heat treatment, cultured cells (at 25°C) were immediately exposed to 40°C for 1h. After heat treatment, cells were collected by infiltration, and total RNAs were immediately isolated and stored in a -80°C freezer for later use. We performed three independent biological replicates and a flask (56 ml) cell suspension represent a biological replicate.

Although heat stress (37–43°C) can induce a new pattern of polypeptide synthesis, expression of HSPs and other thermotolerant proteins is optimal at 40°C for cell cultures (Altschuler and Mascarenhas, 1982; Al-Whaibi, 2011) and would not affect the cells’ viability or cause decreased viability (Key et al., 1981; Rikhvanov et al., 2007). To explore regulatory function of PAL in more protein transcripts, we set the maximum temperature at 40°C. Also, the treatment time at 40°C for both gradual and abrupt heat shock was 1 h. In this paper, we focus on the storage protein which is under different control during heat shock than most other newly synthesized proteins.

### DNA Library Preparation and PacBio Sequencing

**Figure 1:**
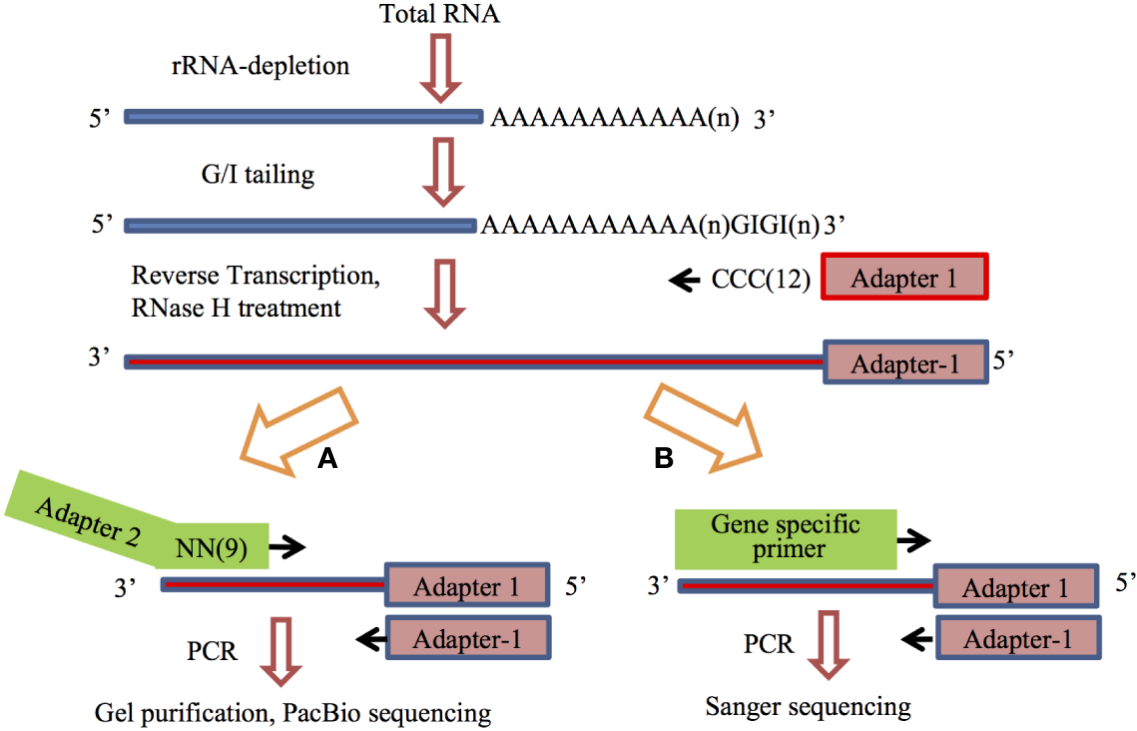
The flowchart of the Assay for Poly(A)-tail Length sequencing. Number or n in the parenthesis indicate repeat of the nucleotide. **(A)** The overall protocol for library construction and sequencing, APAL-seq. **(B)** Experimental design for the confirmation of variation of poly(A) tail length.

The APAL-seq protocol consists of 6 steps, as shown in **Figure 1A**. The protocol variation of poly(A) tail length confirmation is shown in **Figure 1B**. We performed three independent biological replicates. These are briefly described below.

(1) RNA isolation and rRNA depletion. Total RNA was isolated from Arabidopsis cell culture, and rRNA was removed using the Rico-Zero rRNA removal kit (Epicenter, RZPL11016). (2) Attachment of GI tail. RNA samples (0.05 ng–2 μg) were heated at 65°C for 5 min to destroy potential secondary structure at the 3′-end and immediately placed on ice. Subsequent incubation (2 h, 37°C) was carried out after addition of 1,500 units of Yeast Poly(A) Polymerase (Affymetrix), 15 μM GTP, 5 μM ITP, 0.5 μl RNase inhibitor, and 30 µl reaction mixture containing 6 μl 5x Poly(A) polymerase reaction buffer. The reaction was terminated by heating the mixture at 65°C for 10 min, and the RNAs were then purified by NucleoSpin ^®^ RNA Clean-up XS kit (MACHEREY-NAGEL). (3) Production of first strand cDNA *via* GI tailing. GI-added RNAs were mixed with 0.1 μM primer Adapter 1 (**Table S1**) and 10 μM dNTP. The mixture was heated at 65°C for 5 min and then quickly cooled down in an ice bath, followed by a 1 h incubation at 48°C in a thermocycler with 3 μl 10 x RT buffer, 1 μl DTT, 1μl DNase inhibitor and 1μl reverse transcriptase. Ribonuclease H (1 μl, RNase H) was added to the reaction. A continuous incubation was performed at 37°C for 1 h, followed by a 20 min incubation at 65°C to inactivate RNase H. The final reaction products were purified by NucleoSpin^®^ Gel and PCR Clean-up kit. (4) Production of second strand cDNA by random primer PCR. The single-stranded cDNA (100 ng) was incubated with 36 pmol random primer containing nine random nucleotides and the Adapter 2 (**Table S1**) in a thermocycler at 98°C for 3 min. The sample was ramped down to 4°C at a rate of 0.1°C/sec, and then kept in ice. A 30 min incubation at 37°C was performed after mixing the sample with 20 μM dNTP, 3 μl 10 x reaction buffer and 5 units of the Klenow fragment (3’-5’ exo-) enzyme (NEB). The mixture was then incubated at 75°C for 20 min to inactivate the enzyme, followed by purification with NucleoSpin^®^ Gel and PCR Clean-up kit (MACHEREY-NAGEL). Purified sample was diluted in 30 μl RNase-Free Water. (5) PCR amplification. PCR reaction system was composed of 3 μl cDNA, 5 μl of 10x PCR buffer, 3 μl of 50mM MgCl_2_, 200µM dNTPs, 0.5 μl Taq DNA polymerase, and 0.5 μl of 1mM Primer1 (#1553) and Primer2 (#1534) (**Table S1**). The reaction parameters were set up as follows: 2 min at 94°C, 25 cycles of 1 min at 94°C, 30 s at 60°C, 2 min at 72°C and 10 min at 72°C. (6) Purification of the PCR products. Upon agarose gel electrophoresis, gel covering two ranges of the PCR products in 200–500 bp and 500–1,000 bp were isolated by using NucleoSpin^®^ Gel and PCR Clean-up kit. After a quality check by Agilent 2100 Bioanalyzer, the library was ready for sequencing by PacBio sequencing (University of Delaware Genome Facility houses a PacBio RSII Sequencer).

### Bioinformatics Analysis

Analysis of the PacBio sequencing data to search for poly(A) sites was largely similar as detailed shown before (Wu et al., 2011). These are briefly described below.

(1) Identification of poly(A) sites. To identify the poly(A) sites, poly(A) or poly(T) track from the sequences obtained from PacBio sequencing was first identified. A qualified poly(A)/(T) tail must have more than 8 A or T nucleotides in a long sequence. (2) Identification of primers. An appropriate transcript usually had matching or complementary sequence for Primer #1553 and Primer #1534 in the beginning and at the end. To determine potential primer for genes with poly(A)/(T) tail, fuzzy matching was carried out between the first 30 nucleotides at both ends and two primers [Primer 1534 and Primer C#1553 (complement to Primer #1553) or Primer1553 and Primer C#1534]. If matching was more than 70%, it indicated that the beginning and the end of the sequence had correct primer sequences. (3) Identification of Gn or Cn. Length, position and number of C or G nucleotide was determined between the primer and poly(T) or poly(A) tail. Both were used for judgment on rationality of sequence composition. (4) Distinguish barcodes. If sequenced by barcode, only cDNA with complementary sequence to barcode and appropriate position could be contributed to their original source. Following insert-search usually applied when a sequence had primer, poly(A/T) tail and barcode. (5) Identification of insert sequences. For sequences with poly(T) and the primer at the 3’ end, insert sequence was between poly(T) and primer. For sequences with poly(T) but not primer at 3’ ends, insert sequence was between poly(T) and the 3’ end. For sequences with poly(A) and primer at 5’ ends, insert sequence was between poly(A) and the primer. For sequences with poly(A) but not primer in 5’ ends, insert sequence was between poly(A) and the 5’ end. If an insert was shorter than 20 nt, it was discarded. (6) Comparison of insert sequences. Insert sequences were processed with BLAT and Perl script management to identify poly(A) information including position, length etc. The results were compared with updated TAIR10 genome annotation (ftp://ftp.arabidopsis.org/home/tair/Genes/TAIR10_genome_release/) to identify gene and genic region (3’ UTR, CDS, intron, 5’UTR). (7) Variation of poly(A) tail lengths. In order to avoid potential errors introduced by genomic PCR and PacBio sequencing, three genes AT3G24780, AT5G42300, AT5G65220 (**Table S1**) were picked, with variable poly(A) tail lengths (30nt, 60nt, 150nt) by PacBio, and the average poly(A) tail lengths of these three genes are 32, 63, 155 nt by Sanger sequencing three times (**Figure 1**), respectively. These results are consistent with the previous results that the optimized PCR step and PacBio sequencing will not introduce errors to the poly(A) sequence (Clarke et al., 2001).

### Protein Analysis

Precipitated cells after heat treatment, or control, were ground in a precooled mortar with pestle until becoming a fine powder and then transferred to an Eppendorf tube with addition of 1 ml ice-cold lysis buffer (50 mM Tris-HCl, pH 7.5, 10% glycerol, 2% SDS, 25 mM EDTA). Centrifugation was performed for 1 h at top speed at 4°C, followed by gentle removal of tubes from the centrifuge and placement on ice. Supernatant was then transferred to a fresh tube and kept on ice. The amount of protein was quantified by bicinchoninic acid (BCA) Protein Assay kit (Thermo Scientific) based on a standard protocol. Protein band density in Western blot was visualized and quantified using an Odyssey Infrared Imaging System (LI-COR, Lincoln, NE) (Holdorf et al., 2012). We performed three independent biological replicates.

Western blot analysis of HSP70 and HSP17.6C was performed essentially as described (Shen and Shi, 2016). The washed and blocked membrane was incubated with 1:5000 of primary HSP70 antibody (Hsp70/Hsc70 (Plant) Antibody, My BioSource Inc.) or HSP17.6 antibody (Anti-HSP17.6 antibody, Abcam) in blocking buffer overnight at 4°C. After incubation with conjugated secondary antibody, the target proteins were detected by immunoblotting *via* chemiluminescence (Amersham™ ECL™ Prime, GE Healthcare).

## Results

### Accurate Measurement at the Transcriptome Level by the APAL-Seq Protocol

We have developed and optimized a protocol termed APAL-seq (Assay for Poly(A) Tail Length - sequencing) that fulfills the gap between sequencing platforms and library construction by providing a transcriptome level poly(A) tail length measurement. It takes the full advantage of a GI (guanosine and inosine) tailing method and a random primer extension method, effectively reducing starting RNAs to 0.5 ng. It also circumvents ligation, thus making library construction relatively easy and efficient. Finally, it uses PacBio platforms for poly(A) homopolymer sequencing.

In APAL-seq (**Figure 1A**), adapters were added to the 3’ ends of the first strand cDNA by random primer extension, which construct cDNA libraries with very low amounts of total cDNA and short incubation times. Adapter-mediated ligation is the classic method for introducing adapters to the 3’ end of the first strand cDNA. However, this method is slow in product formation and release, thus requiring high levels of templates, large quantities of enzymes and long incubation times (Stark et al., 2006; Zhuang et al., 2012). In addition, APAL-seq can adjust the range of cDNA libraries by changing the amount of random primers present (Hodgson and Fisk, 1987). Illumina sequencing is suitable for determining libraries ranging from 200 to 500 bp and PacBio circular consensus sequencing can measure larger sample sizes with an upper limit of 1000 bp. While both methods, such as TAIL-seq (Zhuang et al., 2012; Chang et al., 2014), were designed for the Illumina platform, APAL-seq could also be used for both sequencing techniques.

GI tailing was used in our protocol. It stabilizes poly(A) tails by adding a limited number (~25) of guanosine and inosine residues to the 3’ end of RNAs with the help of yeast poly(A) polymerase. Unlike traditional oligoguanosine (oligo(G)) tailing (Kusov et al., 1996), GI tailing is less likely to form self-pairing structures that could interrupt normal sequencing process. It also creates an oligo guanosine-inosine tract that can pair with the oligo(C) primer in reverse transcription. Two T residues are usually added to the oligo(C) primer to better match with the poly(A) at the 3’ end of RNAs. The ratio of GTP to ITP in GI tailing resulted in affected product sizes. Two ratios (1:1 and 3:1) of GTP to ITP were used for GI tailing, followed by reverse transcription with a gene (tubulin) specific primer and a universal primer (#1553) (**Table S1**). Sanger sequencing further showed that the sequence of the band perfectly matches with the tubulin gene. Different amounts of the RNA template (0.05 ng, 1 μg and 10 μg) were used in GI tailing. These GI tailing products were then processed in steps including reverse transcription and 2nd cDNA extension. qPCR results demonstrated that 75%, 82%, and 73% of the PCR products were successfully attached by the two adapters. These results show that GI tailing was sensitive when detecting template levels and required a template amount as low as 0.05 ng, which was much lower than that of the adapter-mediated ligation method (Janicke et al., 2012).

The authenticity of PAL was further confirmed with Sanger sequencing by randomly picking three genes with varied PAL for comparison. After PacBio sequencing, AT3G24780, AT5G42300, and AT5G65220 (primers shown in **Table S1**) with variable PAL (30nt, 60nt, 150nt) were chosen. Primers were designed based on the sequencing results and PALs were re-sequenced by Sanger sequencing three times independently. **Figure 1B** shows the experimental design. The average PALs of these three genes detected by Sanger sequencing are 32, 63, and 155 nt. Comparisons made with PacBio sequencing results suggested that the protocol can accurately measure PAL at the transcriptome scale.

### Large-Scale Search of Poly(A) Tail Elongation Upon Heat Stress

**Figure 2:**
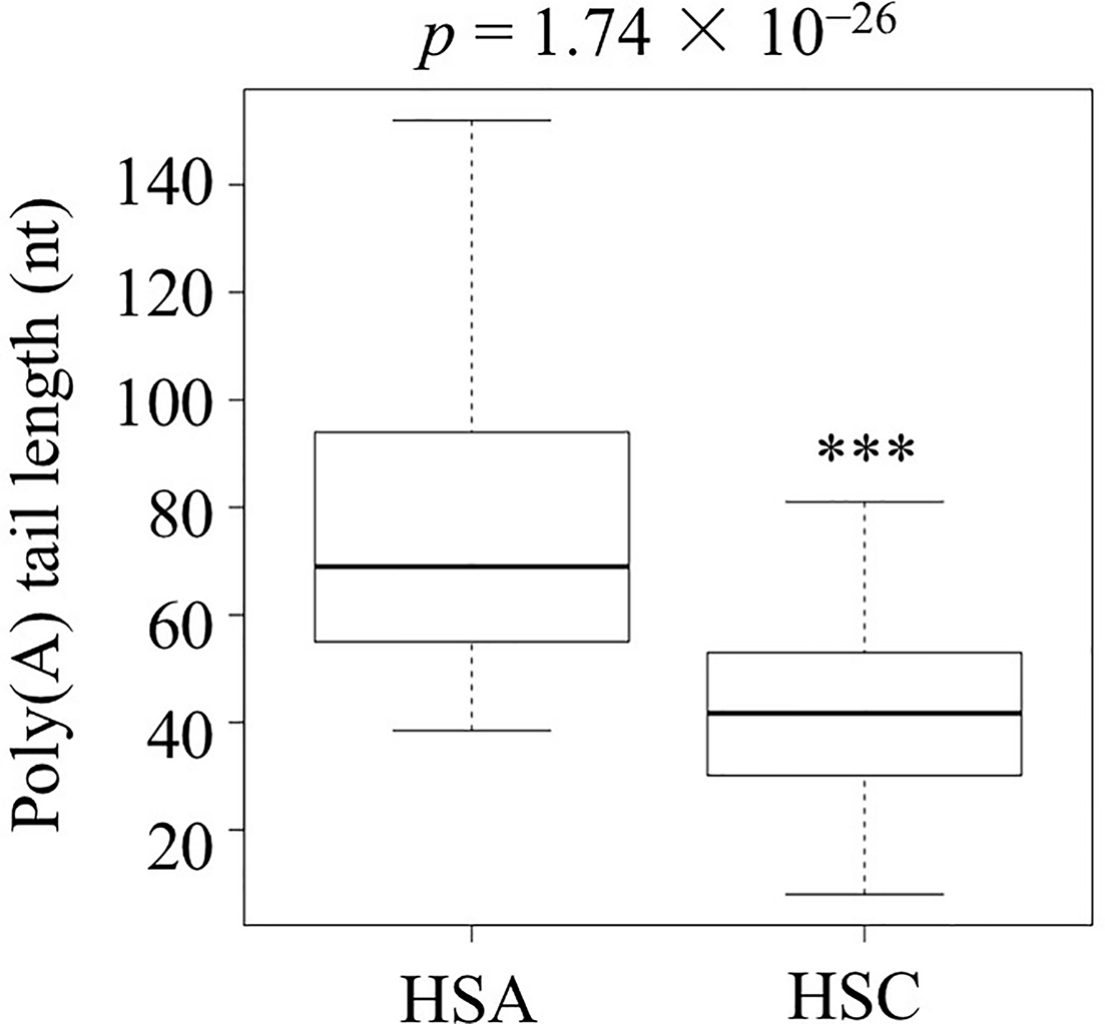
The poly(A) tail length of the 122 transcripts related to heat shock in the abrupt heat shock (HSA) and control (HSC) libraries. Shown is the analysis carried out by three biological replicates. (^***^ *p < *0.001).

After successfully establishing the protocol, RNAs from suspended culture cells under no stress (heat-shock control or HSC) or abrupt stress (heat-shock abrupt or HSA) conditions were isolated. These two APAL-seq libraries were produced and sequenced with PacBio sequencing. Among 13,863 transcripts, sequencing results showed that 2,477 had variable PAL between control and abrupt heat shock and that 1,160 out of 2,477 transcripts had longer PAL under abrupt heat treatment compared to the control. A gene list and the heatmap of GO is provided in **Dataset S1** “2,477 genes with PAL change” and **Figures S1** and **S2**, respectively. Among these 1,160 transcripts, 122 transcripts were identified as involved in thermotolerance or related pathways by GO (Gene Ontology) annotation and shown in **Dataset S1** “1,160 genes with longer PAL”, “122 genes related to heat shock” and **Figure S3**, respectively; the APAL-seq result of these 122 transcripts were shown in dataset packages “HSC” and “HSA” in SRA databases (https://www.ncbi.nlm.nih.gov/sra/PRJNA553644). The average PAL of the 122 genes shows significantly longer PAL in HSA than in the HSC library (*p-*value=1.74 × 10^−26^, **Figure 2**, and **Dataset S1** “122 genes related to heat shock”). PCR libraries included two replicates of the control and abrupt heat stress treatments as well as three replicates of gradual heat stress. The same amount of barcoded cDNA from all libraries were pooled for a second round of PacBio sequencing (**Table S2**). These exercises resulted in the 14 interested transcripts (**Dataset S1** “14 interested genes”) with variable PALs that could be identified from all libraries. These genes were selected for detailed analysis below based on their PAL profiles (primers were shown in **Table S3**).

### Longer Poly(A) Tails Yielded on Some Genes Irrespective of Heat Stress Regime

**Figure 3:**
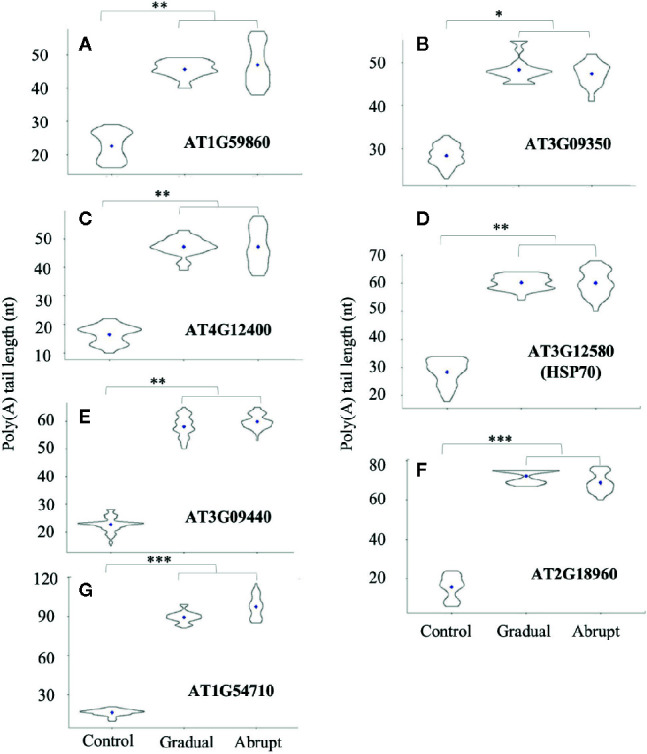
Poly(A) tail length profile (violin plots) of Group one transcripts during heat treatments. The length of poly(A) tails is reflected on the Y-axis, and the distribution range is reflected by the shape and size of the “violin”. The width of the “violin” implies the abundance of transcripts. The diamond in each “violin” indicates the average length of poly(A) tails. Each “violin” shows the distribution range of three biological replicates. (^***^ *p < *0.001, ^**^ *p < *0.01, ^*^ *p < *0.05, ns represents no significant difference). **(A–G)** The change of the poly(A) tail length of different genes after gradual or abrupt heat shock.

Based on the above analysis, transcripts from seven genes that exhibited longer poly(A) tails under either abrupt or gradual heat shock were selected for further study. The PALs of these transcripts were changed from 25 to 40 nt in both gradual and abrupt heat treatments. Five of the seven target genes are related to HSP. **Figure 3A** and **Table S4** showed that PAL of the HSP20-like protein (AT1G59860) transcript increased from 22 to 46 nt upon both gradual and abrupt heat stress. Similar changes were also found on the transcripts of AT3G09350 encoding HSP70-binding protein, from 25 to 49 and 48 nt in gradual and abrupt heat stress (**Figure 3B**, **Table S4**), respectively. AT4G12400 encoding co-chaperones interacting with Hsp90/Hsp70 changed the PAL from 16 to 46 nt in both heat stresses (**Figure 3C**, **Table S4**). HSP70 (AT3G12580) and HSP70-3 (AT3G09440) transcripts increased from 25 to 61 nt upon both gradual and abrupt heat stresses (**Figures 3D, E**, **Table S4**).

Two other transcripts had the same increasing trend of poly(A) tail lengths. One is the AT2G18960 encoding the plasma membrane protein ATPase, which is related to stomata closure in response to drought. The PAL of this gene was around 15 nt in unstressed cells, but it had longer PAL of 75 and 69 nt in gradual and abrupt heat stressed cells, respectively (**Figure 3F**, **Table S4**). The other similar gene is AT1G54710, which encodes a water stress protein. It should be noted that pathways for water and heat stress are interconnected in plants (Al-Whaibi, 2011). The PAL of this transcript was 16 nt, but it had longer PAL of 95 nt in both heat-stressed cells (**Figure 3G**, **Table S4**).

Thus, we conclude that these seven target genes encoding HSP, HSP-related, or other proteins involved in the heat stress pathway were found to have shorter poly(A) tails before heat treatment, but similarly elongated poly(A) tails after both gradual and abrupt heat treatments.

### Lengthening of Poly(A) Tails of Some Genes Based on Severity of Heat Stress

**Figure 4:**
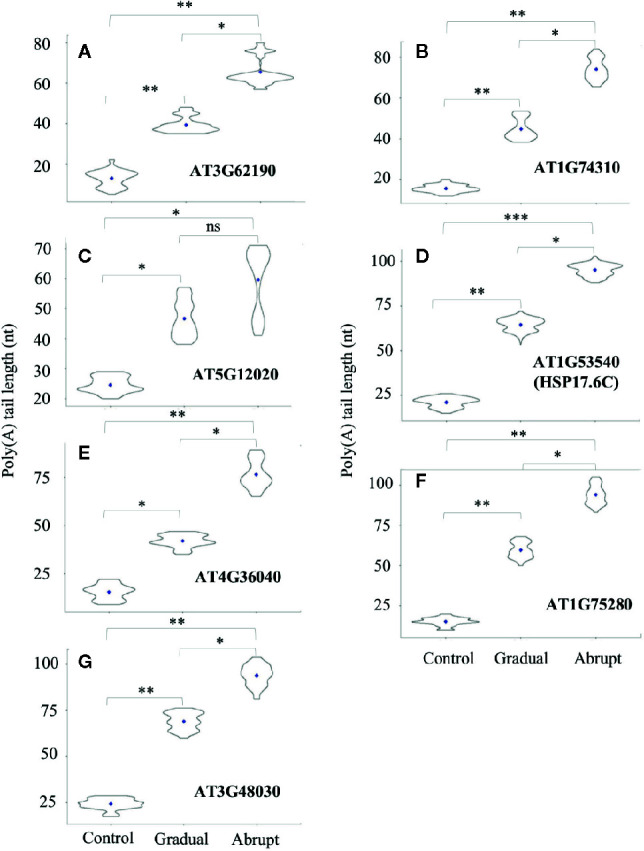
Violin plot of poly(A) tail lengths of Group two transcripts during heat treatments. Refer to **Figure 3** legend for figure annotations. **(A–G)** The change of the poly(A) tail length of different genes after gradual or abrupt heat shock.

While poly(A) tails of several transcripts changed after gradual or abrupt heat treatment, increase of their tail lengths was proportional to the degree of heat stress. As shown in **Figure 4** and **Table S4**, five HSPs or HSP-related genes fell into this category. Poly(A) tails of the HSP40 (AT3G62190) transcript changed from 13 to 40 nt under gradually increasing heat treatment and to 66 nt upon abrupt heat shock. The poly(A) tail of HSP101 (AT1G74310) transcript was 19 nt under unstressed conditions, while its average PAL increased to 56 and 76 nt in gradual and abrupt heat treatments, respectively. The HSP17.6II (AT5G12020) transcript had a poly(A) tail of 24 nt without stress, but it was elongated to 47 nt in gradual treatment and 60 nt in abrupt treatment. The HSP17.6C (AT1G53540) transcript had a poly(A) tail of only 21 nt, but it was lengthened to 65 nt in gradual treatment and 96 nt in abrupt treatment. AT4G36040 encodes a protein which can bind to HSPs. Its PAL was only 15 nt in control, but increased to 42 nt in gradual treatment and 77 nt in abrupt heat shock.

Two more target genes involving the heat shock-related pathway showed the same changes in PAL. One was AT1G75280, which encodes a protein involved in oxidative stress response. Its PAL in control was 15 nt, but it increased to 60 and 94 after gradual and abrupt heat treatments, respectively (**Figure 4F**, **Table S4**). The other was AT3G48030, which encodes a hypoxia-responsive protein. Its poly(A) tail was much longer, at 56 nt and 93 nt, respectively, under gradual and abrupt heat treatments compared to 19 nt under control conditions (**Figure 4G**, **Table S4**).

Thus, poly(A) tail lengthening of some transcripts can reflect the intensity of the stimulus, suggesting, in turn, that polyadenylation can somehow “sense”, or be limited by unknown factors that restrict the elongation of poly(A) tails. Additionally, the group threshold (the PAL gaps between abrupt and gradual heat shocks) among treatments of these 14 target transcripts was calculated (**Table S5**, L6), where transcripts in group 1 (**Figure 3**) had similar PAL between gradual and abrupt heat shocks (threshold ≤1), while transcripts in group 2 (**Figure 4**) had a significant difference in PAL between gradual and abrupt heat shocks (1< threshold <2).

### HSP70 Protein Production Is Induced by the Heat Shock Treatment

**Figure 5:**
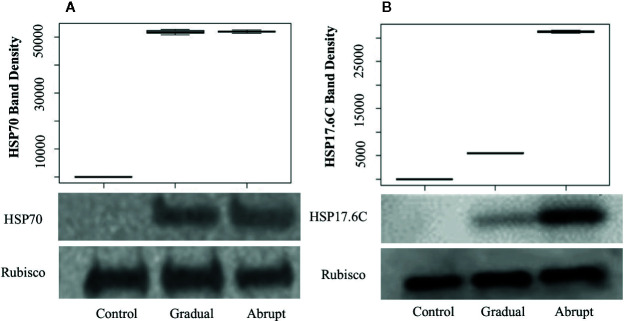
Western blots and band density of HSP70 **(A)** and HSP17.6C **(B)** in *Arabidopsis* cell culture during different heat shock treatments. Gradual: gradual heat treatment; Abrupt: abrupt heat treatment. Rubisco large subunit served as a loading control. Shown is a representative experiment carried out by three biological replicates.

To determine whether the elongation of poly(A) tail indeed contributes to the expression level of proteins, quantitative Western blots were carried out to detect the amount of protein production under heat stress conditions. To ensure that the amount of loading protein among different treatments was equal, the Bicinchoninic acid (BCA, see *Materials and Methods*) Kit was used to quantify the starting amount of the loading proteins and the Rubisco large subunit protein was used as loading control to ensure that the total amount loaded proteins were equal. APAL-seq results showed that poly(A) tail lengths of several genes in group 1 (**Figure 3**, **Table S5**) were elongated similarly whether in gradual or abrupt heat treatment relative to the control. For example, the PAL of HSP70 transcript (At3g12580, **Figure 3D**, **Table S4**) in unstressed cells was only 25 nt, but elongated to ~60 nt in both gradual and abrupt heat stressed cells. Western blot results (**Figure 5A**) showed that the HSP70 protein was only detected in cells treated with gradual and abrupt heat stress, not in unstressed cells. The relative protein band density of HSP70 was 0, 51,800, and 52,010 (relative density measurement; **Figure 5A**), indicating an induction of HSP70 expression from heat stress. On the contrary, the level of the Rubisco protein was similar in both unstressed and stressed cells, with a band density of 765,000, 765,800, and 765,300, respectively. Therefore, similar elongation of PAL occurred in stressed cells, with a similar increasing trend in the expressed protein. Combining the results of poly(A) tail lengths in encoding mRNA (**Figure 3D**), it can therefore be concluded that elongated poly(A) tails are an influential factor in HSP70 protein expression.

### HSP17.6C Induction Is Proportional to the Degree of Heat Stress

APAL-seq results showed that poly(A) tail lengths of several genes were elongated in both gradual and abrupt heat treatments relative to the control. However, PAL was longer in abrupt heat stress compared to gradual heat stress in Group 2 genes (**Figure 4**, **Table S5**). For example, while the HSP17.6C transcript had a short poly(A) tail (20 nt) under unstressed conditions, it elongated (65 nt) in mild heat stress, then PAL was further elongated up to 96 nt in abrupt head stress (**Figure 4D**, **Table S4**). To investigate the contribution of PAL in translation efficiency, protein expression of HSP17.6C was measured. Equal amounts of proteins were loaded to the gel with Rubisco as a control. No HSP17.6C protein was expressed without stress (**Figure 5B**) and while the expression of some HSP17.6C proteins were detected under gradual heat stress, most HSP17.6C proteins were expressed upon abrupt heat stress. The band density of Rubisco on average, as measured by Image Studio Lite Ver software, was 765,000, 765,800, and 765,300, indicating that the loading protein was almost the same. The average band density of HSP17.6C was 0, 5,510, and 31,300 (**Figure 5B**). The band density in the abrupt heat condition is almost 6-fold more than that in gradually increasing heat conditions, suggesting that the amount of HSP17.6C protein induced in abrupt heat condition was significantly increased over that induced by gradual heat shock. Collectively, these results show that the HSP17.6C protein level was proportional to the poly(A) tail length, indicating that the length of the poly(A) tail may contribute to translation efficiency.

## Discussion

### Measuring Poly(A) Length Using APAL-Seq

Current research demonstrates that the length of poly(A) tails in mRNAs is variable and that it determines the fate of the transcript in terms of its stability and ability to be translated. Thus, measuring poly(A) tail length of these transcripts could be used to reflect the state and efficacy of stored or functional mRNA. Early research was mostly built on methods that included G-tailing (Kusov et al., 1996), ligation-mediated poly(A) testing (LM-PAT) (Janicke et al., 2012), and the RNase H assay (Mustroph et al., 2009). These protocols were designed to analyze only one or a few genes, or to rely on the ligation steps, requiring long experimental time, which does take advantage of the Illumina sequencing platform for globally measuring PAL (Chang et al., 2014; Zheng and Tian, 2014; Zhao et al., 2019). However, they can only sequence the library cDNA within a short range (250–350 bp in length). In contrast, APAL-seq circumvents this problem by allowing for the reading of much longer sequences while also avoiding the issue of homopolymer base calling, which is found in the Illumina platform. The PacBio sequencing platform enables the observation of natural DNA synthesis by a DNA polymerase as it occurs. Essentially, PacBio sequencing “eavesdrops” on a single DNA polymerase molecule working in a continuous, processive manner. In this method, PacBio sequencing technology can be used for determining poly(A) tail length because it has been proven to sequence homopolyers, like long stretches of “A”s (Jason Underwood et al., 2012). Poly(A) tail lengths longer than 230 nt cannot be measured by Illumina sequencing at the transcript level as a result of limited sequencing cycles (Chang et al., 2014). In summary, APAL-seq overcomes the issue of longer-tailed mRNAs, as noted above, to explore the role of PAL in gene expression.

### Correlation Between PAL and Translation

Poly(A) tails are important elements in mRNA translation and stability. PAL is a determinant in certain tissue contexts, developmental stages, cell cycle regulation, daily rhythmic oscillations of protein synthesis, or cellular stress (Besse and Ephrussi, 2008; Kojima et al., 2012; Park et al., 2016). Two genome-wide studies reveal a weak correlation between the PAL and translational efficiency of mRNAs in cell culture (Chang et al., 2014; Subtelny et al., 2014). Subtelny et al. observed the association of PAL and translation became less apparent in the non-embryonic cells, which were clearly coupled at early developmental stages in Xenopus and zebra fish. Furthermore, well-expressed transcripts containing relatively short, well-defined tails were found in *Caenorhabditis elegans* (Lima et al., 2017). This attribute appears dependent on translational efficiency, as transcripts enriched for optimal codons and ribosome association had the shortest tail sizes, while non-coding RNAs retained long tails. It suggests that instead there might be an optimal tail size that results from a shortening process they refer to as pruning, which inspired this work.

Although the studies challenge the longstanding idea that longer poly(A) tails promote mRNA stability and translation (Goldstrohm and Wickens, 2008; Weill et al., 2012; Wahle and Winkler, 2013; Jalkanen et al., 2014), strong relationships between PAL and mRNA translatability may still emerge under certain conditions. Sheets and Wickens suggested that differences in PAL may contribute to quantitative differences in translational stimulation, with longer poly(A) tails having a stronger stimulatory effect (Sheets et al., 1994; Sheets et al., 1995). This can likely be explained by the longer poly(A) tail which can facilitate translation initiation by promoting the circularization of RNA molecules into a closed-loop shape (Sachs and Varani, 2000), thus improving translation efficiency. Several transcriptome-wide studies revealed that PAL becomes transiently associated with translation efficiency in specific cellular contexts, such as in early embryonic development or during cell cycles (Subtelny et al., 2014; Eichhorn et al., 2016; Park et al., 2016). During ER stress, cap-dependent translation is rapidly and globally repressed (Ron, 2002), which raises the possibility that poly(A) tails are also dynamically regulated in ER stress response. PAL shortening suppresses Pabpc1 mRNA translation in mature cardiomyocytes, thereby reducing overall protein synthesis rates in the adult heart (Chorghade et al., 2017). All these studies involve cellular contexts where the posttranscriptional regulatory landscape is drastically altered, which suggests that translation and poly(A) length may become directly linked (Woo et al., 2018).

### Regulation of PAL During Heat Shock-Induced Damage

It was reported that the poly(A) tail is the major determinant of posttranscriptional mechanisms (Abaza and Gebauer, 2008; Hershey et al., 2012; Weill et al., 2012). Increase in PAL during heat stress has been observed previously. In *Drosophila*, the poly(A) tails of HSP32 and HSP70 elongate during heat stress (Dellavalle et al., 1994). In *Schizosaccharomyces pombe*, severe heat shock leads to the accumulation of bulk poly(A)+ RNA in nuclei (Tani et al., 1996). The poly(A) tails of HSP21 transcripts during abrupt heat stress were 15 - 60 nt longer compared to poly(A) tails under gradual heat stress (Osteryoung et al., 1993). It is therefore reasonable to speculate that PAL, as a regulatory effector, similar to other types of upstream host stress response mechanisms, plays an important role in reactivity to the stress. However, since only limited information is available on whole genome changes in PAL during heat stress, the present study addresses this very question. Taking advantage of PacBio sequencing technology, variations in PAL were determined under heat stress on the genomic level. Poly(A) tails from 14 genes closely involved in heat shock, or crosstalk, pathways were changed by heat stress. These genes had shorter PAL (25 nt) in unstressed cells relative to those under gradual or abrupt heat stress (40 to 90 nt). Thus, we have, for the first time, investigated heat-response changes in PAL at the genomic level. Results suggest that the elongation of poly(A) tail may be a general response mechanism of plants to heat stress.

As essential molecular chaperones or proteases responsible for heat stress, HSPs in plants can prevent damage from protein misfolding through interactions with protein substrates. Our study indicated two possible mechanisms underlying poly(A) tail length-mediated regulation of HSPs. First, the translation of HSPs can only be initiated by elongated poly(A) tail under heat stress conditions. Therefore, it is plausible that many HSP70 family proteins or HSP70-related proteins may be regulated this way. HSP70 family proteins are evolutionarily conserved and commonly expressed in higher plants based on translational templates from 14 genes (Boorstein et al., 1994; Sung et al., 2001; Zhang et al., 2008). It has been proved that expression of a subset of HSP70 is stimulated under heat stress, preventing the aggregation of denatured proteins (Sheffield et al., 1990) and the refolding of stress-denatured proteins (Glover and Lindquist, 1998; Goloubinoff et al., 1999). However, transcriptional mechanisms underlying HSP70 regulation remain unclear. Our results demonstrate that both abrupt and gradual heat treatments could induce accumulation of two HSP70 transcripts (HSP70 and cytosolic Hsp70-HSP70-3) and two HSP70-related protein transcripts (HSP70-binding protein and HSP70-co-chaperones) in *Arabidopsis*. Interestingly, these HSP70 transcripts all experienced repolyadenylation (longer PAL) with significantly increased translational expression during heat treatments.

Another possible mechanism underlying poly(A) tail length-mediated regulation of HSPs involves HSP translation which depends on a poly(A) tail length relative to heat stress severity. In this case, our findings suggest that PAL was dramatically longer under abrupt heat stress when compared to gradual heat stress. HSPs or related proteins include HSP40, HSP101, HSP17.6II, and HSP17.6C. The PAL of HSP17.6C was found to be proportional to the severity of heat stress, leading to a 6-fold dramatic increase in the expression of HSP17.6C during abrupt heat stress compared to gradual heat stress. We noticed that two small heat shock proteins (sHsps), HSP17.6C, and HSP17.6II, behaved in the same way. Mechanisms underlying HSP-mediated protection are not fully understood in living cells. However, it can be speculated that it involves chaperone functions by protecting proteins from irreversible denaturation (Al-Whaibi, 2011). This protection by HSP17.6C family proteins is achieved *via* up-regulation of HSP17.6C proteins in abrupt heat treatment. These results are consistent with previous research (Weill et al., 2012), suggesting that the longer PAL might be positively associated with the translation levels of HSPs. Intriguingly, it was also uncovered that the gradual HS and abrupt HS are two different treatments which may lead to different translational functions. The study sets the stage for elucidating the rapid regulation of longer PAL in lower molecular weight HSPs upon abrupt HS and probing the general roles of PAL in translation.

Regulation of PAL in plants might contribute to cellular processes when other stress conditions come into play. For example, our results showed that the PAL of 4 genes (AT1G54710, AT2G18960, AT1G75280, and AT3G48030) varied during heat treatment and that their established biological functions primarily involve cellular responses to water, drought, as well as oxidative and hypoxic stress, respectively. It would also benefit the study of other stress responses where translation contributes to gene regulation under non-stress and dehydration stress conditions like in *Arabidopsis* as reported (Kawaguchi et al., 2004). Considering possible crosstalk that arises when cells respond to multiple environmental stresses (Al-Whaibi, 2011; Ely et al., 2014). It is plausible that the above-mentioned transcripts are housekeeping genes responsible for preventing primary and secondary damage to cells in higher plants (Al-Whaibi, 2011), which in turn, indicates the possible role of PAL in helping plants to survive under such multiple environmental stresses.

Furthermore, increasing evidence illustrates that the *HSP* genes are significant for normal plant development and responsive to many other forms of stresses (Lin et al., 2001; Kim and Hwang, 2015). For example, the gene family of *HSP70* and *HSP17.6C*, which are of special interest, is essential to plant growth and environmental responses. *Arabidopsis* mutation of *HSP70-16* led to a significant reduction in seed setting rate, caused by failed flower opening, abnormal floral organ formation, and impaired fertilization (Chen et al., 2019). Plants with *HSP17.6A* overexpression could survive longer than the wild-type upon salt stress, where the transgenic plants had high At-*HSP17.6A* transcript and protein levels (Sun et al., 2001).

## Conclusions

The increase in PAL is indeed linked to the increased production of stable proteins upon heat stress. It highlights the relevance of the differential translation regulation of PAL in the establishment of plant stress response, e.g. heat stress. The study described herein provides powerful means to further unravel the functions of the PAL in gene regulation under physiological and pathological conditions in plants. Dynamic control of PAL could serve as an adaptive role in stimulating a beneficial form of genes expression that may be physiologically advantageous.

## Data Availability Statement

All datasets generated and analyzed for this study are included in the manuscript and supplementary files or shown in public databases from SRA (https://www.ncbi.nlm.nih.gov/sra/PRJNA553644).

## Author Contributions

QL, JW, and XuW conceived the plan. QL and YH supervised the project. JW and XuW performed the experiments. XiW performed PacBio data analysis. XuW, JW, and QL wrote the manuscript. QL agrees to serve as the author responsible for contact and ensures communication. All authors contributed to the article and approved the submitted version.

## Funding

The project was funded, in part, by a grant from the National Key R&D Project of China (2016YFE0108800), by the U.S. National Science Foundation (IOS-154173), both to QL. XuW was an awardee of the China Scholarship Council (CSC) for a scholarship under the State Scholarship Fund to pursue her study at Western University of Health Sciences.

## Conflict of Interest

The authors declare that the research was conducted in the absence of any commercial or financial relationships that could be construed as a potential conflict of interest.
