# Supplementary material for: Heat Shock Responsive Gene Expression Modulated by mRNA Poly(A) Tail Length
Source: Front Plant Sci. 2020 Aug 14;11:1255. doi: 10.3389/fpls.2020.01255 (PMC7456977; doi:10.3389/fpls.2020.01255)
Supplement: Supplementary file 1 [file DataSheet_1.docx]

Supplementary Materials

**Supplemental Table 1.** The Primers used for APAL-seq library construction and validations

| **Primers** | **Sequence (5' --- 3')** | **Purpose** |
| --- | --- | --- |
| 1553 | ACACGACGCTCTTCCGATCT | PCR primers for 1^st^ round of PacBio sequencing |
| 1534 | CTGAACCGCTCTTCCGATCT |  |
| AT3G24780 | CGGATTACAAGGTGATTGTGAG | Variation of PAL |
| AT5G42300 | GAGCTTTACTACAACTAGGCC |  |
| AT5G65220 | GCAGAAGAAGCAGCTGAAGCTG C |  |
| Adapter 1 | ACACGACGCTCTTCCGATCTCCCCCCCCCCCCTT | For making cDNA Libraries |
| Adapter - 1 | ACACGACGCTCTTCCGATCT |  |
| Adapter 2 | AGATCGGAAGAGCGGTTCAGNNNNNNNNN |  |

**Supplemental Table 2.** Primers and barcode sequences for library production

| **Libraries** | **Replicates** | **Sequence (5' --- 3')** |
| --- | --- | --- |
| Gradual | Replicate 1 | ACACGACGCTCTTCCGATCTNNACAGTGCCCCCCCCCCCCCTT |
| Control | Replicate 2 | ACACGACGCTCTTCCGATCTNNATCACGCCCCCCCCCCCCTT |
| Gradual |  | ACACGACGCTCTTCCGATCTNNCTAGCTCCCCCCCCCCCCTT |
| Abrupt |  | ACACGACGCTCTTCCGATCTNNCAGATCCCCCCCCCCCCCTT |
| Control | Replicate 3 | ACACGACGCTCTTCCGATCTNNTACAGCCCCCCCCCCCCCCTT |
| Gradual |  | ACACGACGCTCTTCCGATCTNNACTGATCCCCCCCCCCCCCTT |
| Abrupt |  | ACACGACGCTCTTCCGATCTNNCGATGTCCCCCCCCCCCCCCTT |

Note: The nucleotides underlined are barcodes.

**Supplemental Table 3.** Target genes (14) with their gene specific primers

| **Target Gene IDs** | **Primer sequence (5' --- 3')** | **Purpose** |
| --- | --- | --- |
| AT1G54710 | ACTCACTGATGATCTTTAATACCCTG | For making libraries of the target genes for 2^nd^ round of PacBio sequencing |
| AT4G12400 | ACAAGAGCATATGAAGAACCCA |  |
| AT2G18960 | AGGGAAGAGAACTCTTGTGTATT |  |
| AT1G59860 | CGGAGGCGCACGTGTTCAAG |  |
| AT3G09440 | GTCGACTAATAACTTTCTCTC |  |
| AT3G09350 | CGATGAGAA ACT GAGGCA GC |  |
| AT3G12580 | CTTGTTCTTAGTTTTATCTTTC |  |
| AT4G36040 | GGAACTAGTGTGCTAACATAC' |  |
| AT1G75280 | CACAAGTGTTGACGAGTATCT |  |
| AT3G48030 | ATCGTTGTATCTTGATCAATTG |  |
| AT3G62190 | GCCTAGAGTCTGGTGATG |  |
| AT1G74310 | GGTGAAGAAGATGAGGATCG |  |
| AT5G12020 | GGTTACTGTTCCGAAACTTCCTC |  |
| AT1G53540 | TGTGTGATGGTAGTGAAATAATTG |  |

**S****upplemental Table 4.** The average poly(A) tail length (nt) and copy number of 14 transcripts in treatments.

|  | **Treatments** | | | | | |
| --- | --- | --- | --- | --- | --- | --- |
| **Gene IDs** | **Control** | | **Gradual heat shock** | | **Abrupt heat shock** | |
|  | **Ave.** **PAL.** | **Copy No.** | **Ave. PAL.** | **Copy No.** | **Ave. PAL.** | **Copy No.** |
| AT1G59860 | 22 | 208 | 46 | 181 | 47 | 166 |
| AT1G54710 | 16 | 128 | 90 | 179 | 98 | 210 |
| AT3G12580 | 25 | 180 | 61 | 173 | 60 | 158 |
| AT4G12400 | 16 | 129 | 47 | 126 | 46 | 158 |
| AT3G09350 | 25 | 139 | 49 | 104 | 48 | 134 |
| AT2G18960 | 15 | 97 | 75 | 161 | 69 | 153 |
| AT3G09440 | 23 | 349 | 58 | 247 | 60 | 255 |
| AT4G36040 | 15 | 125 | 42 | 122 | 77 | 124 |
| AT3G62190 | 13 | 128 | 40 | 105 | 66 | 148 |
| AT1G75280 | 15 | 173 | 60 | 201 | 94 | 197 |
| AT1G74310 | 19 | 170 | 56 | 175 | 76 | 161 |
| AT3G48030 | 19 | 160 | 56 | 146 | 93 | 166 |
| AT5G12020 | 24 | 126 | 47 | 204 | 60 | 86 |
| AT1G53540 | 21 | 150 | 65 | 172 | 96 | 169 |

**Supplemental Table 5.** The group threshold among treatments of the 14 target transcripts.

| **Threshold** | **Line** | **L1** | **L2** | **L3 = L2 / L1** | **L4** | **L5 = L4 / L1** | **L6 = L5 / L3** |
| --- | --- | --- | --- | --- | --- | --- | --- |
|  | **Gene IDs** | **Control**  **Ave. PAL.** | **Gradual**  **Ave. PAL.** | **Ratio (Gradual / Control)** | **Abrupt**  **Ave. PAL.** | **Ratio (Abrupt / Control)** | **Threshold** |
| **Group 1:** | AT1G59860 | 22 | 46 | 2.09 | 47 | 2.14 | 1.02 |
|  | AT1G54710 | 16 | 90 | 5.63 | 98 | 6.13 | 1.09 |
|  | AT3G12580 | 25 | 61 | 2.44 | 60 | 2.40 | 0.98 |
|  | AT4G12400 | 16 | 47 | 2.94 | 46 | 2.88 | 0.98 |
|  | AT3G09350 | 25 | 49 | 1.96 | 48 | 1.92 | 0.98 |
|  | AT2G18960 | 15 | 75 | 5.00 | 69 | 4.60 | 0.92 |
|  | AT3G09440 | 23 | 58 | 2.52 | 60 | 2.61 | 1.03 |
| **Group 2:** | AT4G36040 | 15 | 42 | 2.8 | 77 | 5.13 | 1.83 |
|  | AT3G62190 | 13 | 40 | 3.08 | 66 | 5.08 | 1.65 |
|  | AT1G75280 | 15 | 60 | 4.00 | 94 | 6.27 | 1.57 |
|  | AT1G74310 | 19 | 56 | 2.95 | 76 | 4.00 | 1.36 |
|  | AT3G48030 | 19 | 56 | 2.95 | 93 | 4.89 | 1.66 |
|  | AT5G12020 | 24 | 47 | 1.96 | 60 | 2.50 | 1.28 |
|  | AT1G53540 | 21 | 65 | 3.10 | 96 | 4.57 | 1.48 |


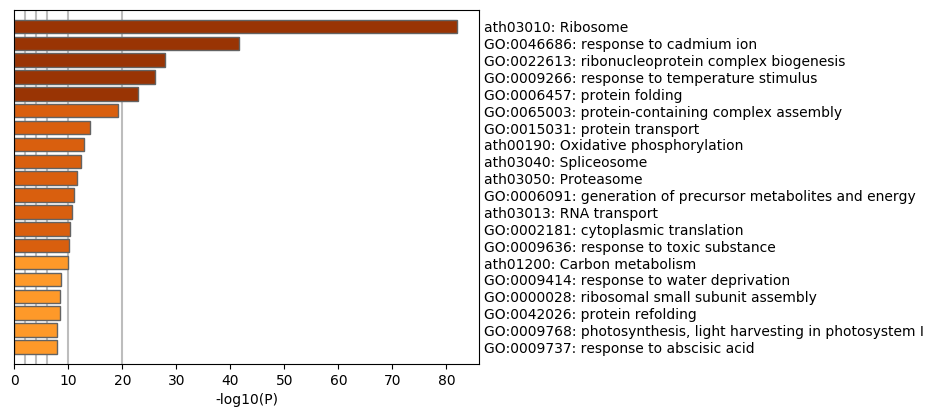


**Supplemental Figure 1.** Heatmap GO of 2477 transcripts whose PAL were changed


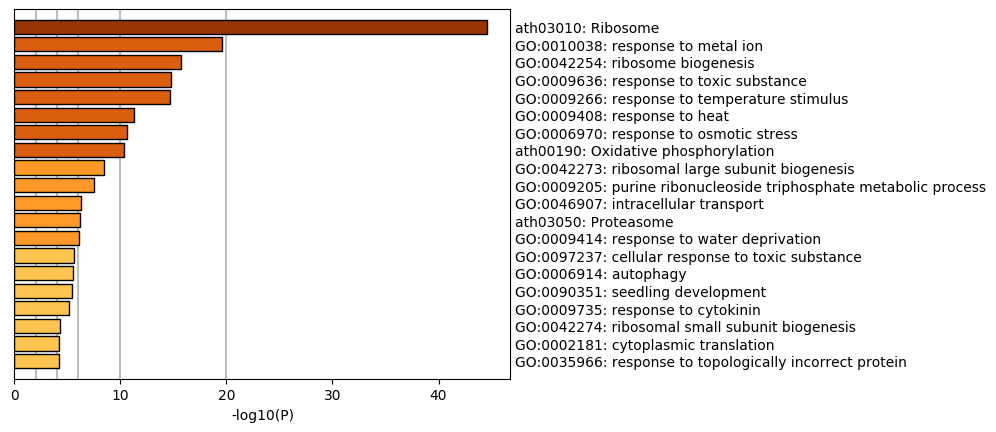


**Supplemental Figure 2.** Heatmap GO of 1160 transcripts whose PAL were longer in HSA


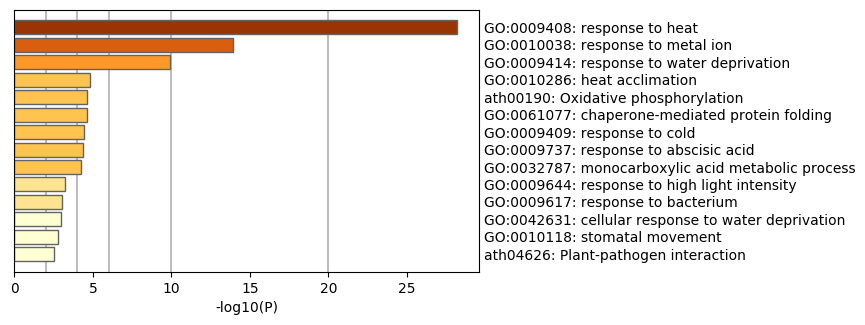


**Supplemental Figure 3.** Heatmap GO of 122 transcripts which are involved in thermotolerance or related pathways
